# Evaluation of the effect of statin treatment on intracranial atherosclerotic plaques using magnetic resonance vessel wall imaging: a case series

**DOI:** 10.3389/fneur.2025.1539212

**Published:** 2025-03-17

**Authors:** Ke Yang, Pengyu Zhou, Yuting Wang, Bin Huang

**Affiliations:** ^1^Department of Radiology, Sichuan Provincial People’s Hospital, School of Medicine, University of Electronic Science and Technology of China, Chengdu, China; ^2^Department of Magnetic Resonance Imaging, Fuwai Hospital, National Center for Cardiovascular Diseases, Chinese Academy of Medical Sciences and Peking Union Medical College, Beijing, China; ^3^Department of Neurology, Sichuan Provincial People’s Hospital, School of Medicine, University of Electronic Science and Technology of China, Chengdu, China

**Keywords:** MRI, vessel wall imaging, intracranial atherosclerosis, statins, efficacy assessment, case reports

## Abstract

**Introduction:**

Intracranial artery stenosis highly increases the recurrence risk of transient ischemic attack and ischemic stroke, especially in Chinese patients. Patients with intracranial atherrotic disease (ICAD) should be actively treated with risk factor control, such as lipid management. This report discusses vessel wall MRI (VW-MRI) to evaluate plaque in-situ changes in four patients with ICAD after anti-lipid therapy of statins.

**Case report:**

Four patients with ischemic stroke and ICAD were prospectively enrolled. VW-MRI and serum low-density lipoprotein cholesterol (LDL-C) were assessed at baseline and follow-up (at least 11–12 months). All patients received statins throughout the study. Compared with baseline, the LDL-C decreased in one case, the length of basilar artery plaque and the overall plaque enhancement segment were shortened, and the plaque thickness was reduced, indicating that the plaque tended to regress. In the second case, LDL-C increased after one year compared with baseline, along with upgraded plaque enhancement and new intraplaque hemorrhage, indicating plaque progression. After 2.5 years, LDL-C decreased significantly, while VW-MRI changes were minimal. LDL-C increased in the third case, but VW-MRI indicated plaque regression. In the fourth case, LDL-C decreased significantly, and the degree of basilar artery plaque stenosis was reduced. However, plaque enhancement upgraded, and intraplaque hemorrhage increased, indicating plaque progression.

**Conclusion:**

VW-MRI can monitor the in-situ changes of plaques after lipid-lowering therapy with statins, provide key information that is difficult to reflect in systemic serological lipid indices like LDL-C, and help identify cases that are not responsive to current anti-lipid therapy.

## 1 Introduction

Cerebral ischemic stroke causes a heavy burden on society due to its high recurrence rate and disability rate (1). Approximately 46% of ischemic stroke patients in China are caused by intracranial atherosclerotic disease (ICAD) (2). Decreased hemodynamic perfusion induced by ICAD and unstable rupture of responsible plaques are associated with ischemic stroke events. Therefore, in addition to using traditional assessment to evaluate arterial narrowing, it is particularly important to evaluate the characteristics of ICAD plaques.

Magnetic resonance vessel wall imaging (vessel wall MRI, VW-MRI) is a non-invasive vascular imaging method that has been widely used in clinical intracranial vessels since the publication of the expert consensus by the American Society of Neuroradiology in 2017 (3). The technical core of VW-MRI is the black blood technology, which suppresses the intravascular blood signal to allow for good signal contrast between the arterial wall and the blood, allowing for high-resolution visual observation of the intracranial arterial wall structure, clarifying the distribution of atherosclerotic plaques relative to the lumen, judging the characteristics of plaques, determining the degree of vessel remodeling, etc.

Both the Chinese Guidelines for Secondary Prevention of Ischemic Stroke and Transient Ischemic Attack 2022 and the Chinese Scientific Statement on the Long-term Management of Blood Lipids in Patients with Ischemic Stroke and transient ischemic attacks recommend that ICAD patients should actively adopt risk factor control therapy, including lipid management (4, 5).

Some studies have shown that statins can lower low-density lipoprotein cholesterol (LDL-C) and enhance the stability of coronary and carotid plaques, preventing plaque rupture (6, 7). However, the effect of LDL-C control on ICAD could be different due to the thick inner elastic lamina and lack of outer elastic lamina in the intracranial artery (8), and there are fewer related reports (9–14), in contrast with the internal carotid artery. This study prospectively collected and reported 4 cases of ICAD-induced ischemic stroke and conducted a long-term statin therapy based on the Guidelines 2022 to explore the clinical value of using VW-MRI to evaluate the efficacy of statin anti-lipid treatment on ICAD plaques (4).

## 2 Case report

### 2.1 Case 1

**Figure 1:**
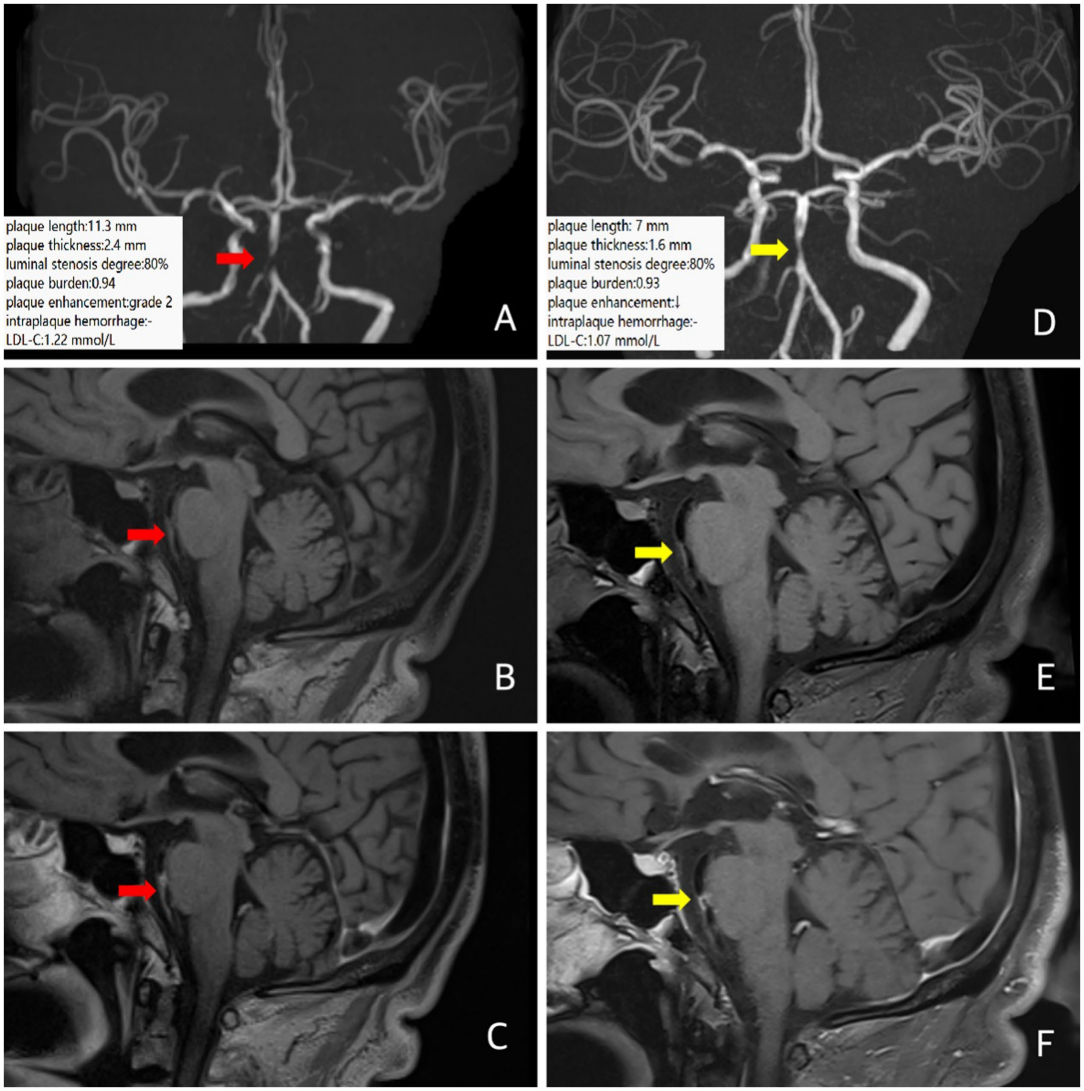
In case 1, patient at baseline (red arrow) and follow-up images 12 months later (yellow arrow). Three-dimensional time-of-flight cerebral magnetic resonance angiography showed significant stenosis in the middle segment of the basilar artery **(A,D)**. Coronal T1 plain scan weighted imaging **(B,E)** and enhanced imaging **(C,F)** in whole brain magnetic resonance imaging using different inversion angles to optimize contrast showed multiple atherosclerotic plaque formation in the basilar artery with obvious stenosis in the middle lumen and obvious plaque enhancement. Follow-up intracranial VW-MRI showed that compared with baseline, the length of the plaques was shortened, the plaque thickness was attenuated and there was no significant change in luminal stenosis, plaque burden, or plaque enhancement. Still, the overall enhancement segment was shortened, suggesting that the plaque tended to be stable. The LDL-C levels decreased.

A 64-year-old male patient presented to the hospital with a 3-h history of sudden left-sided limb and facial numbness. He had a history of diabetes and had been receiving long-term insulin therapy with good glycemic control. He had a clinical diagnosis of ICAD. The brain MRI showed a fresh infarction lesion on the right side of the pons. MRA suggested severe stenosis of the basilar artery near the middle segment. Intracranial VW-MRI showed multiple atherosclerotic plaque formation with obvious stenosis of the middle segment of the basilar artery. The plaque length was 11.3 mm, the plaque thickness was 2.4 mm, the degree of luminal stenosis [luminal stenosis degree = (1-luminal area at stenosis / normal luminal area) × 100%] was about 80%, and the plaque burden [plaque burden = (1− luminal area at stenosis / total vascular area)], was about 0.94. The plaque enhancement was grade 2 (Grade 0 means no enhancement, with plaque signal intensity ≤ adjacent normal intracranial artery wall signal intensity. Grade 1 means mild to moderate enhancement, pituitary infundibulum enhancement > plaque signal intensity > normal artery wall signal intensity. Grade 2 means obvious enhancement, with plaque signal intensity ≥ pituitary infundibulum enhancement) (15), suggesting plaque instability (Figures 1A–C). Following the neurologist’s instructions, the patient took a long-term high-intensive rosuvastatin (20 mg/d) treatment. After 12 months, follow-up VW-MRI showed the plaque length was 7 mm, the plaque thickness was 1.6 mm, the luminal stenosis was about 80%, the plaque burden was about 0.93, the plaque enhancement degree was still grade 2, and the overall plaque enhancement segment was shortened (Figures 1D–F), indicating that the plaque tended to be stable. LDL-C decreased from 1.22 mmol/L at baseline to 1.07 mmol/L at follow-up, consistent with the change of image of image findings. There was no recurrence of stroke or TIA in the following time.

### 2.2 Case 2

**Figure 2:**
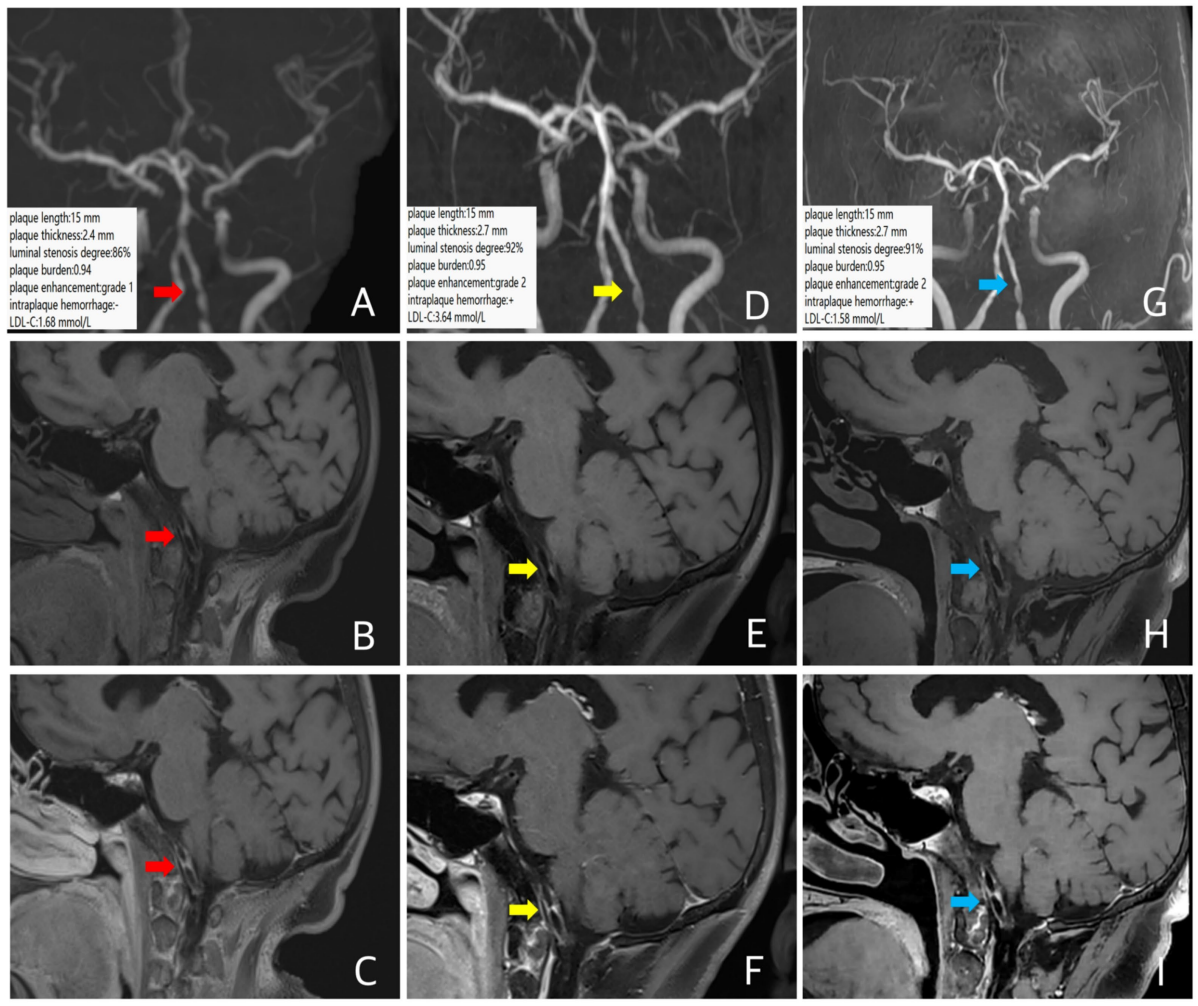
In case 2, patient at baseline (red arrow), follow-up images 11 months later (yellow arrow), and follow-up images 30 months later (blue arrow). Three-dimensional time-of-flight cerebral magnetic resonance angiography showed significant stenosis of the intracranial segment of the left vertebral artery **(A,D,G)**. Coronal T1 plain scan weighted imaging **(B,E,H)** and enhanced imaging **(C,F,I)** in whole brain magnetic resonance imaging using different inversion angles to optimize contrast showed multiple atherosclerotic plaque formation in the left vertebral artery plaque with obvious stenosis and obvious plaque enhancement. The 11-month follow-up intracranial VW-MRI showed no significant changes in plaque length and plaque thickness compared with baseline, a slight increase in the luminal stenosis and the plaque burden, a new intraplaque hemorrhage occurred, and the plaque enhancement upgrades **(D–F)**, indicating a progressive tendency in the plaque. Which LDL-C level is elevated. At 30 months, intracranial VW-MRI showed no significant changes in plaque compared with the second examination **(G–I)**, in which LDL-C levels decreased.

A 67-year-old female patient presented with dizziness for 1 month. MRA revealed left vertebral artery stenosis. She had a history of diabetes, with poor glycemic control, and a clinical diagnosis of ICAD. Intracranial VW-MRI showed atherosclerotic plaque formation in the intracranial segment of the left vertebral artery with significant luminal stenosis, with a plaque length of 15 mm, a plaque thickness of 2.4 mm, a luminal stenosis of about 86%, a plaque burden of about 0.94, and the plaque enhancement was grade 1, indicating some instability (Figures 2A–C). During the follow-up period, the patient took a moderate-intensity to high-intensity rosuvastatin (10–20 mg/d) treatment intermittently as poor complicance. A follow-up VW-MRI was performed 11 months later. Compared with the baseline examination, the plaque length was 15 mm, the plaque thickness was 2.7 mm, the luminal stenosis was about 92%, the plaque burden was about 0.95, the fresh intraplaque hemorrhage (The presence of fresh IPH was identified as >150% signal relative to nearby medial pterygoid muscles on precontrast T1-weighted images) (16) was detected and the plaque enhancement was elevated to grade 2 (Figures 2D–F), suggesting a trend of plaque progression, indicating that the anti-lipid therapy was not effective. The LDL-C increased from 1.68 mmol/L at baseline to 3.64 mmol/L at the point of follow-up, consistent with the image findings. A third intracranial VM-MRI at 30 months follow-up was performed. Compared with the second scan, there were no significant changes in plaque length, plaque thickness (15 mm and 2.7 mm, respectively), luminal stenosis and plaque burden (about 91% and 0.95, respectively), intraplaque hemorrhage and plaque enhancement (Figures 2G–I), indicating no significant change in plaque characteristics. The LDL-C decreased from 3.64 mmol/L at the second time to 1.58 mmol/L at the third time.

### 2.3 Case 3

**Figure 3:**
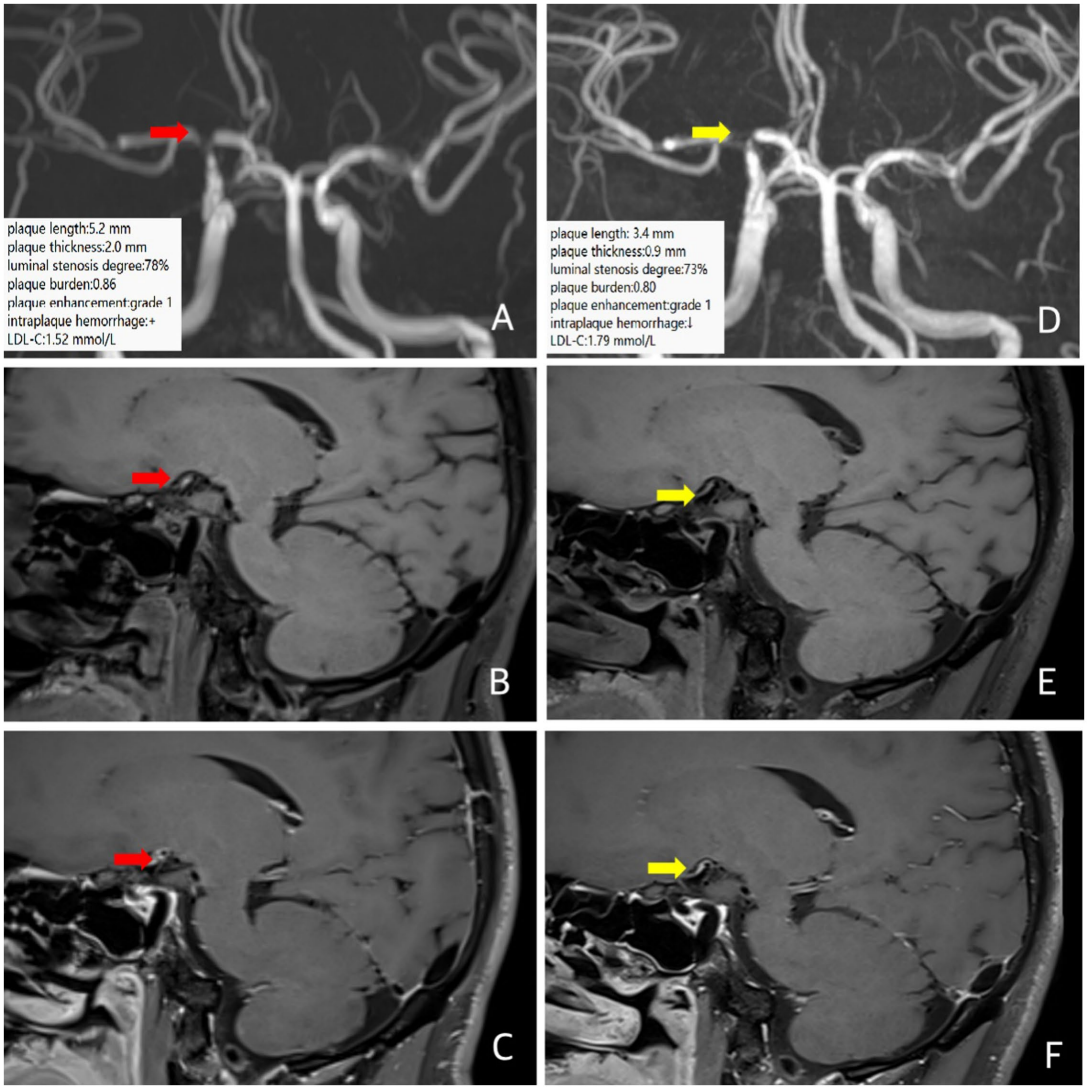
In case 3, the patient is at baseline (red arrow) and follow-up images 12 months later (yellow arrow). Three-dimensional time-of-flight cerebral magnetic resonance angiography showed significant stenosis of the right internal carotid artery communicating segment **(A,D)**. Coronal T1 plain scan weighted imaging **(B,E)** and enhanced imaging **(C,F)** in whole brain magnetic resonance imaging using different inversion angles to optimize contrast showed atherosclerotic plaque was formed in the right internal carotid artery communicating segment, with obvious stenosis of the lumen, intraplaque hemorrhage (T1 signal significantly higher than the surrounding muscle signal) and obvious plaque enhancement. Follow-up intracranial VW-MRI showed a shorter plaque length and a lower plaque thickness than the baseline, a slight reduction in luminal stenosis and plaque burden, and a weakening of signals in intraplaque hemorrhage, suggesting that the plaque is receding and stabilizing. The LDL-C level is elevated.

A 37-year-old male patient presented with a transient left-hand numbness and slurred speech for 4 h. He had a history of hypertension. The clinical diagnosis was ICAD. MRI and MRA at an outside hospital showed multiple infarctions in the right frontal, parietal, temporal, and occipital lobes, as well as severe stenosis of the right internal carotid artery communicating segment. Baseline intracranial VW-MRI showed atherosclerotic plaque formation in the right internal carotid artery communicating segment with severe focal stenosis, the plaque length was 5.2 mm, the plaque thickness was 2.0 mm, the degree of luminal stenosis was about 78%, the plaque burden was about 0.86, with possible intraplaque hemorrhage, and the plaque enhancement was grade 1 (Figures 3A–C), suggesting plaque instability. The patient took a high-intensity rosuvastatin (20 mg/d) treatment. A follow-up VW-MRI was performed 11 months later. VW-MRI showed that the plaque length was 3.4 mm, the plaque thickness was 0.9 mm, the luminal stenosis was about 73%, the plaque burden was about 0.80, and the intraplaque hemorrhage seemed weakened, indicating that the plaque tended to be stabilized and regress (Figures 3D–F). On the contrary, the LDL-C elevated from 1.52 mmol/L at baseline to 1.79 mmol/L at follow-up, indicating a clinical mismatch.

### 2.4 Case 4

**Figure 4:**
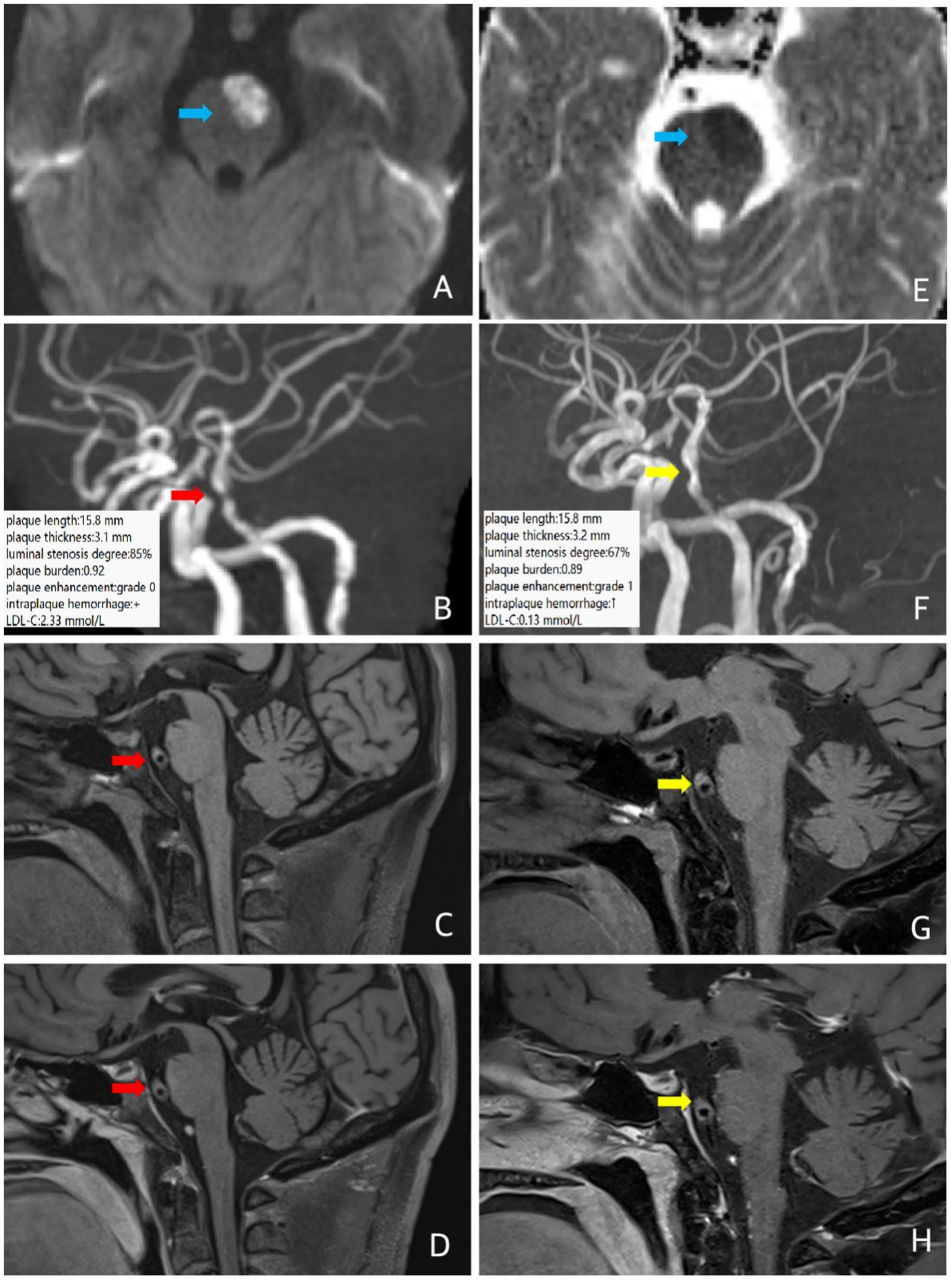
In case 4, patient at baseline (red arrow) and follow-up images 12 months later (yellow arrow). Three-dimensional time-of-flight cerebral magnetic resonance angiography showed the basal artery is tortuous and long, and the middle part is narrow. Coronal T1 plain scan weighted imaging **(C,G)** and enhanced imaging **(D,H)** in whole brain magnetic resonance imaging using different inversion angles to optimize contrast showed multiple atherosclerotic plaques were found in the basilar artery, especially in the middle segment, obvious stenosis of the lumen, eccentric thickening of the left wall of the basilar artery, and suspected intraplaque hemorrhage (T1 signal was higher than that of the temporal muscle nearby). There was no obvious enhancement of the plaque. The follow-up intracranial VW-MRI showed no significant changes in plaque length, plaque thickness, and plaque burden compared to baseline, a slight increase in luminal stenosis, plaque enhancement upgrades, and increased TI high signal **(C,G)**, suggesting plaque progression. Which LDL-C level decreased significantly. In case 4, diffusion sequence (blue arrow) showed limited diffusion in the left anterior portion of the pons, increased signal on DWI **(A)** and decreased signal on ADC **(E)**, suggesting a fresh infarct.

A 57-year-old male patient was admitted to the hospital with sudden right limb weakness and was clinically diagnosed with ICAD. MRI and MRA showed acute infarction in the left anterior part of the pon and severe stenosis of the middle segment of the basilar artery. He had a history of hypertension for more than 20 years, a history of diabetes mellitus for 15 years, and poor glycemic control. Baseline intracranial VW-MRI showed atherosclerotic plaque formation in the basilar artery wall with severe focal luminal stenosis. The plaque length was 15.8 mm, the plaque thickness was 3.1 mm, the degree of luminal stenosis was about 85%, the plaque burden was about 0.92, and the plaque enhancement was grade 0, with possible intraplaque hemorrhage, suggesting some instability (Figure 4). The patient took a long-term high-intensity rosuvastatin (20 mg/d) treatment, and received a subcutaneous injection of the proprotein convertase subtilisin/kexin type 9 (PCSK9) inhibitor (evolocumab) 140 mg, once every two weeks. Follow-up VW-MRI after 12 months showed that the plaque length was 15.8 mm, the plaque thickness was 3.2 mm, the degree of luminal stenosis was about 67%, the plaque burden was about 0.89, the intraplaque hemorrhage seemed increased, and the plaque enhancement elevated to grade 1, indicating plaque progression (Figure 4). However, the LDL-C decreased from 2.33 mmol/L at baseline to 0.13 mmol/L at the end point of follow-up, indicating a clinical mismatch.

## 3 Discussion

Compared with traditional luminal imaging techniques, VW-MRI can directly show the structural characteristics and some pathophysiological changes of plaques while assessing the degree of vascular luminal stenosis (17–19). Studies of carotid plaques have shown that lowering LDL-C can stabilize plaques by removing cholesterol from plaques, decreasing lipid core content, inducing plaque shrinkage, and even increasing plaque calcification content (6, 20). VW-MRI showed good agreement between the composition of intracranial plaques and histology and the observer (21), which provides a theoretical basis for evaluating intracranial plaque efficacy using this imaging technique.

Statins remain the first-line treatment for controlling serum cholesterol levels and lowering LDL-C. A meta-analysis of randomized clinical trials of statins showed that a 1 mmol/L reduction in LDL-C was associated with a 22% reduction in the incidence of major vascular events. Reducing LDL-C to <1.8 mmol/L (normal reference value is 1.0 to 3.3 mmol/L) or less than 50 percent of baseline may be more effective in preventing ischemic stroke recurrence and improving clinical outcomes (22). LDL-C levels have also been found to be associated with plaque changes. Although LDL-C seems a good serologic marker reflecting the efficacy of statins, a study (23) showed that the burden of ischemic stroke attributable to either elevated apolipoprotein B (apoB) or non-high-density lipoprotein (non-HDL) cholesterol is higher than that attributable to elevated low-density lipoprotein (LDL) cholesterol. Cases of worsening plaques during follow-up despite the decrease of LDL-C have been reported, indicating the importance of evaluating the in-situ changes of plaques (24).

The meta-analysis of Zhou et al. (25) showed that the optimal re-evaluating time of VWI for the carotid artery was more than 12 months, and the intracranial plaque changes were shown as early as 11–12 months. We found that the length and thickness of plaque were still good indicators, which was consistent with the study of Huang et al. (26) showed that in patients treated with statins for >6 months (*n* = 31), plaque length, wall thickness, plaque burden, luminal stenosis, and plaque enhancement were significantly reduced. Moreover, we found that plaque enhancement and intraplaque hemorrhage may be more readily observable than other plaque information, suggesting that they could be sensitive indicators of plaque changes. For example, after taking the booster dose of statin antilipid for a long time, the LDL-C level of case 1 decreased by about 13%, within 1.8 mmol/L, and there was no significant change in the degree of luminal stenosis and the plaque burden, while the plaque enhancement segment was shortened, indicating that the plaque tended to be stable. In case 4, PCSK9 evolocumab was added to the booster-dose statin anti-lipid, and the LDL-C level decreased significantly. Although the degree of luminal stenosis and the plaque burden was reduced, the intraplaque hemorrhage increased and the plaque enhancement escalated, suggesting plaque progression. Further research is needed to increase the sample size and validate the results with consideration of individual differences.

It is worth noting that a clinical mismatch between LDL-C changes and plaque changes occurred in some cases. In case 2, after intermittent anti-lipid therapy with standard to intensive doses of statins, the second follow-up showed that although the LDL-C level decreased significantly, the plaque changes were not obvious on VW-MRI. In case 3, although the LDL-C level remained within 1.8 mmol/L (but tended to increase) after long-term intensive dose statin anti-lipid therapy, VW-MRI showed that the plaque had a tendency to regress or had already regressed, which preceded the decline in LDL-C level. In addition, case 4 added PCSK9 evolocumab to intensification-dose statin-based antilipid therapy, and there was also a mismatch between LDL-C changes and VW-MRI reflecting plaque progression. In recent years, some scholars have reported a mismatch similar to case 4. In a long-term follow-up of a 47-year-old man with acute occipital lobe infarction caused by ICAD, Xiao et al. (24) found that despite the decrease in LDL-C levels, one of the plaques had an increase in stenosis and enhancement rate from baseline at a 3-month VW-MRI follow-up, suggesting early identification of patients who did not respond well to medications. Chung et al. (13) also found a similar situation in the follow-up study. They used VW-MRI to examine 77 patients with ICAD-induced acute ischemic stroke found that after 6 months of treatment with an intensive dose of statin (atorvastatin 40–80 mg/d or rosuvastatin 20 mg/d), 35 percent of patients still had no change in the volume and degree of ICAD plaque enhancement (defined as less than 25% change, non-responders) even increased (defined as an increase of more than 25%, progression). The decrease in LDL-C was smaller in nonresponders and progressors (−47.3 ± 38.2 versus −74.8 ± 34.1, *p* = 0.002) compared with good responders, suggesting that nonresponders and progressors may be insensitive to statins.

The study (27) showed that the role of elevated serum LDL-C in the progression of plaque enhancement in offenders was not linear, LDL was independently associated with ischemic events in Grade-1 enhancement plaques (OR 6.778, 95%CI 2.122–21.649, *p* = 0.001). In patients with Grade-2 enhancement plaques, however, LDL was not associated with ischemic events; in contrast, Neutrophil/Lymphocyte ratio was independently associated with ischemic events caused by Grade-2 enhancement plaques (OR 2.188, 95%CI 1.209–3.961, *p* = 0.010). In addition, a study (26) has shown that the efficacy of statins may be influenced by some factors, including individual age, BMI, high blood pressure, blood glucose, and duration of statin therapy. The research (28) indicates there are differences in the characteristics of plaque enhancement and stroke patterns between atherosclerosis in the anterior and posterior circulation. This may be one of the potential mechanisms explaining the differing effects of lipid-lowering therapy. The factors responsible for the phenomenon of mismatch between LDL-C changes and plaque changes are worth further investigation.

Randomized clinical trials in recent years have demonstrated that the addition of newer lipid-lowering agents such as the selective enteric cholesterol absorption inhibitors ezetimibe or PCSK9 evolocumab to statin therapy can further reduce LDL-C levels and reduce the risk of ischemic stroke recurrence, providing new treatment options for patients who are allergic to statins or are insensitive to response (29, 30). Based on the clinical mismatch between LDL-C changes and plaque changes, doctors can monitor the changes in ICAD plaques through VW-MRI, especially by the plaque enhancement and intraplaque hemorrhage, which is consistent with the results of Lou et al. (31), early identification of patients who are not sensitive to the efficacy of current drugs and help to formulate new treatment options or evaluate participation in clinical trials of other endovascular treatments to reduce the risk of stroke recurrence.

## 4 Conclusion

As indicated in these four cases of ICAD, VW-MRI could directly monitor the in-situ changes of intracranial vessel wall plaques after anti-lipid treatment of statins, provide key information that would be otherwise difficult to be timely reflected by serum marker LDL-C, and help tailor the treatment strategy by identifying cases that are not sensitive to current anti-lipid regimen by the time of one year. The mismatch between the plaque changes on intracranial VM-MRI and the LDL-C changes is worthy of further cohort studies.

## Data Availability

The datasets presented in this article are not readily available because of ethical and privacy restrictions. Requests to access the datasets should be directed to the corresponding author/s.
